# A Survey of Substance Misuse Prevalence and Management in Patients Admitted to a Male Acute Ward, a Female Acute Ward, and a Male Psychiatric Intensive Care Unit in KMPT

**DOI:** 10.1192/bjo.2025.10526

**Published:** 2025-06-20

**Authors:** Faizaan Syed, Ifeoma Neye-Akogo, Rubayat Jesmin, Shantala Satisha

**Affiliations:** Kent and Medway NHS Trust, Dartford, United Kingdom

## Abstract

**Aims:** Substance misuse is a common comorbidity in severe mental illness, contributing to increased morbidity and poorer clinical outcomes. Effective management requires accurate documentation and structured interventions. However, existing practices in psychiatric inpatient care are often inconsistent, necessitating a thorough evaluation to inform service development.

Aims were to assess the prevalence of substance misuse and evaluate its documentation and management among patients admitted to Willow Suite Psychiatric Intensive Care Unit (PICU), Pinewood (male acute ward), and Cherrywood (female acute ward) at Littlebrook Hospital between June and July 2024.

Hypothesis: Substance misuse is prevalent among psychiatric inpatients and is under-documented and sub-optimally managed across acute and PICU settings at Littlebrook Hospital.

**Methods:** A retrospective review of clinical records for 96 consecutive admissions (Willow Suite PICU: n=28, Pinewood: n=35, Cherrywood: n=33) was conducted. Data collected included demographics, diagnoses, substance misuse history, documentation practices, and management interventions such as care planning, multidisciplinary team (MDT) discussions, CPA meetings, referrals to specialist services, and psychoeducation.

**Results:** Of the 96 patients, 53% (n=51) had a history of substance misuse, with current misuse documented in 33% (n=32). PICU had the highest prevalence (60%, n=17), followed by Pinewood (51%, n=18) and Cherrywood (48%, n=16). Cannabis was the most frequently reported substance (100% in Willow Suite, 29% in Pinewood, 33% in Cherrywood), followed by cocaine (45%), alcohol (14%), and opiates (10%). Polysubstance use was noted in 47% of Willow Suite patients, 45% in Pinewood, and 44% in Cherrywood.

Across the wards, substance misuse was documented in 34% of core assessments and 42% of progress notes. Care plans addressed substance misuse in only 12% of cases, while MDT reviews and CPA meetings discussed it in 22% and 13%, respectively. Referrals to external substance misuse services were rare (3%, n=3). Psychoeducation was offered to 15% (n=15) of patients.

**Conclusion:** Substance misuse is highly prevalent among inpatients, yet its management remains inconsistent. Gaps in documentation and limited referrals to specialist services indicate the need for improved screening, structured care planning, and closer collaboration with external agencies. These findings highlight an urgent need for targeted service improvements to enhance care for this vulnerable population.

